# Automatic extraction and measurement of individual trees from mobile laser scanning point clouds of forests

**DOI:** 10.1093/aob/mcab087

**Published:** 2021-07-07

**Authors:** Anne Bienert, Louis Georgi, Matthias Kunz, Goddert von Oheimb, Hans-Gerd Maas

**Affiliations:** 1 Institute of Photogrammetry and Remote Sensing, Technische Universität Dresden, Dresden, Germany; 2 Institute of General Ecology and Environmental Protection, Technische Universität Dresden, Tharandt, Germany

**Keywords:** Mobile laser scanning, tree segmentation, forest inventory, ecology, tree crowns, temperate forest, crown architecture, forest structure

## Abstract

**Background and Aims:**

In addition to terrestrial laser scanning (TLS), mobile laser scanning (MLS) is increasingly arousing interest as a technique which provides valuable 3-D data for various applications in forest research. Using mobile platforms, the 3-D recording of large forest areas is carried out within a short space of time. Vegetation structure is described by millions of 3-D points which show an accuracy in the millimetre range and offer a powerful basis for automated vegetation modelling. The successful extraction of single trees from the point cloud is essential for further evaluations and modelling at the individual-tree level, such as volume determination, quantitative structure modelling or local neighbourhood analyses. However, high-precision automated tree segmentation is challenging, and has so far mostly been performed using elaborate interactive segmentation methods.

**Methods:**

Here, we present a novel segmentation algorithm to automatically segment trees in MLS point clouds, applying distance adaptivity as a function of trajectory. In addition, tree parameters are determined simultaneously. In our validation study, we used a total of 825 trees from ten sample plots to compare the data of trees segmented from MLS data with manual inventory parameters and parameters derived from semi-automatic TLS segmentation.

**Key Results:**

The tree detection rate reached 96 % on average for trees with distances up to 45 m from the trajectory. Trees were almost completely segmented up to a distance of about 30 m from the MLS trajectory. The accuracy of tree parameters was similar for MLS-segmented and TLS-segmented trees.

**Conclusions:**

Besides plot characteristics, the detection rate of trees in MLS data strongly depends on the distance to the travelled track. The algorithm presented here facilitates the acquisition of important tree parameters from MLS data, as an area-wide automated derivation can be accomplished in a very short time.

## INTRODUCTION

Forests cover a total area of roughly 4 billion hectares globally (FAO, 2020). Given global deforestation and the dramatic decline in biodiversity, monitoring these changes is of great importance (FAO and UNEP, 2020). Hence, there is an urgent need to increase the areas that are under continuous monitoring. For many years, forest inventories have relied on networks of plots with a fixed radius and a systematic (or random) sampling scheme covering larger areas. The data sampled on these plots include a variety of measured tree parameters, at the level of individual trees, their neighbourhoods and the entire stand. Diameter at breast height (DBH), tree height and position play a crucial role here. One possibility for a higher spatial, temporal and contactless monitoring of forests and plants is offered by corridor-wise mobile laser scanning (MLS).

Modern measuring techniques allow for comprehensive and high-resolution 3-D imaging of complex objects such as trees of different ages (thin vs. thick stem and branch structures of young and old trees, respectively) within a very short time. Over the last decade, terrestrial laser scanning (TLS) became established for the monitoring of forested areas and plants. Such TLS systems capture structures with sub-centimetre accuracy in single-scan or multiple-scan modes. In forests, however, TLS range is often limited due to occlusion by trunks, crowns and the understorey vegetation. Coverage of larger areas can be achieved by MLS that may entail vehicle-mounted laser scanners (Liang *et al*., 2016), off-road backpack solutions (Kukko *et al*., 2012; Liang *et al*., 2014) or hand-held scanners (Bauwens *et al*., 2016; Cabo *et al*., 2018), for example. This adds the aspect of movement along a track (trajectory) to static 3-D data acquisition methods. MLS systems consist of one or more laser scanners in combination with a positioning [global navigation satellite system (GNSS)] and orientation system [inertial navigation system (INS)]. The movement of the scanning platform creates a homogeneous point cloud whose density is strongly dependent on motion speed and scan rate, and typically has a similar point density to TLS point clouds. The acquisition of TLS scans, typically in a stop-and-go mode, produces an inhomogeneous point distribution compared with MLS point clouds (Y. Wang *et al*., 2019*a*).

The costs of TLS and MLS systems have fallen due to recent technical developments; hence a low-cost laser scanner might soon be considered part of the standard data recording equipment in forests (P. Wang *et al*., 2019). This increases the demand for algorithms and software to cope with the large quantity of data and for reliable and fast derivation of forest and, specifically, individual tree parameters. The basis of the latter is fully automatic tree detection. Tree detection algorithms show detection rates that are highly correlated to the density of the stand (Liang *et al*., 2016). Previous studies achieved detection rates between 73 % and 100 % in TLS datasets (Maas *et al*., 2008; Murphy *et al*., 2010; Liang and Hyyppä, 2013; Olofsson *et al*., 2014; Burt *et al*., 2019). Moreover, satisfactory accuracy at the spatial scale of sample plots has been achieved in the automatic determination of those tree parameters, such as DBH and tree height, which are relevant for forest inventories. Nevertheless, the accuracy of the main tree parameters depends to a high degree on the application used. Algorithms that fit geometric shapes, such as least squares fitting of circles, ellipses and cylinders, as well as Hough transformations on horizontal point cloud clusters, have been established to determine stem diameter (Simonse *et al*., 2003; Aschoff *et al*., 2004; Bienert *et al*., 2006; Henning and Radtke, 2006; Maas *et al*., 2008). The DBHs determined in TLS datasets show root mean squared errors (RMSEs) of 1.17 and 1.22 cm for a random Hough transform (Liu *et al*., 2018) and an RMSE between 1.48 and 3.7 cm (Aschoff and Spiecker, 2003; Maas *et al*., 2008; Tansey *et al*., 2009; Calders *et al*., 2015), using a least squares circle fitting. Yet, in comparison, the least squares circle fitting proved to be the most suitable method (Tansey *et al*., 2009). Using MLS data, Čerňava *et al.* (2017) determined the DBHs of 682 trees obtained using different methods and compared them with manually measured values. They report RMSEs between 2.65 and 5.57 cm depending on the stem diameter estimation method. In dense forest stands, tree crown detection with TLS systems is always error prone due to occlusion. Above-canopy data could be used to complement the terrestrial viewpoints as shown in Bienert *et al.* (2010), with above-canopy scans from towers or with intracanopy scans acquired using a drone. Wang *et al.* (2021) present intra- and above-canopy drone flights and compare them with ground-based TLS scans. The dense point clouds in the upper crown area have a geometric accuracy comparable with those from MLS data and have an RMSE of the DBH of 2–4 cm and of the stem curve of 4–7 cm.

Analyses of biomass growth or the competitive tree interactions at the local neighbourhood scale require the characterization of the crown architecture, which can be obtained from the 3-D point clouds. Interlocking branches and contact between adjacent crowns, however, are often a challenge with respect to single tree delineation. Manual crown segmentation in the original point cloud is a complicated task that requires manpower and is very time-consuming (up to several hours for a plot), but sometimes results in highly accurate crown delineation as presented in Lin *et al.* (2010), Seidel *et al.* (2011), Metz *et al.* (2013) and Kunz *et al.* (2019). After all, the result of manual segmentation is highly dependent on the operator, the tree density and the intermixing of adjacent crowns. Automated segmentation algorithms (e.g. Raumonen *et al*., 2013; Hackenberg *et al*., 2015*a*; Trochta *et al*., 2017; Burt *et al*., 2019) have the advantage that segmentation of trees is fast and objective, but this is often at the expense of the accuracy of crown delineation. Semi-automatic segmentation methods are conceivable where manual cleaning of missing or wrongly assigned branches is carried out after automatic tree segmentation in a suitable user interface (e.g. Georgi *et al*., 2018). Furthermore, current TLS tree segmentation algorithms require refinement in order to perform well on low-resolution MLS point clouds.

The range and density of the MLS point cloud depend on various factors. These can be divided into two rough categories: (1) stand characteristics, in particular stand density, tree species and age structure of the trees, as well as density and height of the understorey vegetation; and (2) technical features of the Mobile Mapping System, in particular scan rate, as well as the speed of the mobile platform and the precision of trajectory determination. A crucial limitation of MLS point clouds is still the registration accuracy, due to bad satellite visibility below canopy, and to the drift of the inertial measurement unit (IMU) as trajectory lengths increase. The leaf-off season when the understorey vegetation is also less dense provides the most favourable conditions for measurements in deciduous stands.

Tree segmentation from 3-D point clouds of dense forest stands is a challenging task. The first important step before segmentation is the definition of seed points, which is usually accompanied by tree detection. Depending on the dataset, a distinction can be made between top-down approaches (usually airborne data sets: segmentation starting from the top of the canopy) and approaches starting from the trunk (usually ground-based datasets). The simplest segmentation method is to cut out the tree points using a vertical cylinder. This is placed at the rooting position of the tree with a radius depending on the trunk diameter (Maas *et al*., 2008; Schilling *et al*., 2011). However, the correctness of the crown segmentation is affected by the cylinder radius, with the risk of cropping the crown to a simple circular shape (if the radius is smaller than the real crown radius) that might also contain branches from neighbouring tree crowns.

Algorithms for full tree and tree crown segmentation from MLS data are only available for urban trees. Urban environments are usually characterized by a single-row arrangement of roadside trees parallel to the MLS system trajectory and thus provide a clear view of the trunk and crown space. Urban trees mostly stand isolated and allow for the detection and tracking of the tree stems and the branches (Jaakkola *et al*., 2010; Liang *et al*., 2014). Wu *et al.* (2013) separate adjacent trees on the basis of horizontal voxel layers, excluding objects that do not represent trees (facades, cars, etc.). The differentiation of neighbouring crown parts is ultimately performed using a modified region growing algorithm that accounts for distances. Wang *et al.* (2018) modified this algorithm and applied it to airborne laser scanning (ALS), TLS, and MLS data using a group of urban roadside trees. Sensor-specific input parameters such as cuboid size and minimum tree diameter have to be defined.

The goal of our study was to present a novel segmentation algorithm for MLS datasets and to investigate its quality and accuracy for processing a large MLS dataset of a natural mixed forest. Our new algorithm can be considered as an extension of existing segmentation algorithms with the novelty that it is able to (1) detect tree positions and segment trees by applying a distance adaptiveness for clustering and region growing to overcome point inhomogeneities in MLS data; and (2) simultaneously determine tree and, specifically, crown parameters of the segmented trees. To our knowledge, this study is the first to perform fully automatic segmentation and parameter derivation from MLS datasets of natural forest stands. The study addresses two research questions. (1) How does the scan distance from the MLS device affect automated tree segmentation? (2) How does the quality of the automated tree segmentation influence the derivation of forest inventory parameters?

## Materials and methods

### Study area

The study area is located in the Lauerholz Forest (53.88°N, 10.74°E) in Northern Germany. This is a natural mixed forest with an area of 880 ha on flat terrain (maximum 15 m difference in elevation). The forest comprises stand ages of up to 200 years (see Appendix Table A1). A large proportion of the Lauerholz Forest is a mixed deciduous forest dominated by the tree species *Fagus sylvatica* L., *Quercus robur* L., *Carpinus betulus* L., *Fraxinus excelsior* L., *Acer pseudoplatanus* L., *Acer platanoides* L., *Prunus avium* L. and *Betula pendula* Roth. In addition, coniferous species are admixed, in particular *Pinus sylvestris* L., *Picea abies* (L.) H. Karst., *Larix decidua* Mill. and *Pseudotsuga menziesii* (Mirbel) Franco.

### MLS campaign

**Fig. 1.**
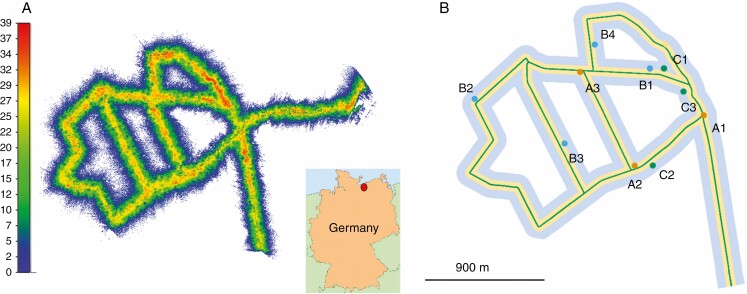
MLS data recording in the Lauerholz Forest, Northern Germany. (A) MLS data of scanner 1 (thinned point cloud, showing every 15th point) with location map; colours indicate the canopy height; (B) MLS trajectory along forest tracks with buffer zones (yellow, 35 m; light blue, 100 m) and indication of the location of the ten sample plots. The colour symbolizes the distance of the plot centre to the trajectory: orange up to 20 m; blue 20 m up to 40 m; green 40 m up to 60 m.

The MLS data were recorded in a measurement campaign in March 2017 under leaf-off conditions using a RIEGL VMX-250 Mobile Mapping System (RIEGL, 2012). This system consisted of two time-of-flight RIEGL VQ-250 laser scanners as well as an integrated IMU and GNSS equipment. A van served as the platform, with the Mobile Mapping System mounted on its roof at a height of 2 m. Forest corridors along forest tracks were captured twice by driving in both directions to reduce the potential effects of shadowing (Fig. 1). Overall, the MLS trajectory had a length of 20 km (10 km one way), and data were captured up to 80 m into the forest on each side of the forest tracks. Due to the one-sided orientation of the mobile laser scanners to the trees along forest tracks, a sector of almost 180° of a trunk surface can be achieved under optimal conditions (full coverage, no occlusion). A first visual analysis showed point cloud segments of stems at a distance of 80 m to the right and left of the forest track, which will allow tree detection. Within a scan rate of 300 kHz (per scanner) and at a speed of 10–15 km h^–1^, an area of almost 160 ha (scanned twice) was recorded within 100 min, with a total of 3.73 billion laser points and 150 GBytes of raw point cloud data. The four MLS point clouds (scanner 1 and 2 in both directions) were registered and a common MLS point cloud was used for further processing. The registration algorithm used works iteratively, with the point cloud of scanner 1 from one direction serving as reference data. The other three point clouds (scanner 1 back, scanner 2 out and back) were co-registered with the iterative closest point (ICP) in an iterative manner using short time intervals of 2 s. The algorithm used has not yet been published.

### TLS campaign

**Fig. 2.**
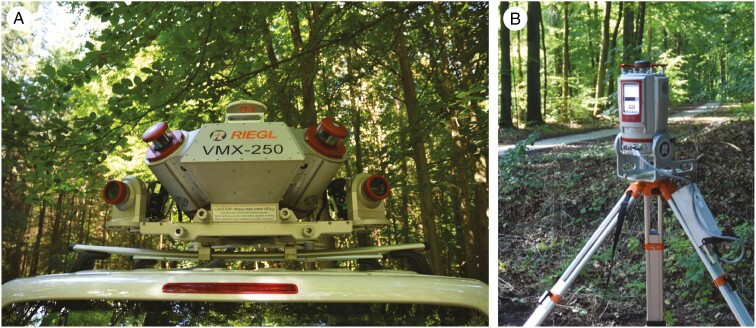
RIEGL VMX-250 Mobile Mapping System (A) and RIEGL VZ400-i (B). The photos were taken during a leaf-on campaign.

TLS measurements were carried out at the same time as the MLS data recording in March 2017 using a RIEGL VZ-400i terrestrial laser scanner. Figure 2 shows the RIEGL VMX-250 Mobile Mapping System and the RIEGL VZ-400i terrestrial laser scanner used for the scanning campaigns. Each sample plot was scanned from five scanner positions in a multiple scan mode, one in the centre of the plots (placed at an inventory point) and the other four positions spread in the four cardinal directions, each 23–25 m from the centre. The angular resolution was set at 0.04°. At the centre position, the scanner was tilted by 90° to overcome the limitation of the panoramic field of view. At the other four positions, the scanner was tilted by 30°. The instrument height was 1.30 m. All TLS point clouds were co-registered with the registration tools ‘Automatic Registration 2’ and ‘Multi Station Adjustment’ of RIEGL RiSCAN Pro 2.6.1 to the central scanner position. This resulted in a registration accuracy between 2.2 and 2.7 mm.

### Validation study on ten sample plots

In order to compare the results of the MLS segmentation with a suitable reference dataset we selected ten plots (Fig. 1B) within the study area for which high-resolution TLS data were available. The location of the plots was based on the inventory sampling scheme of manual inventory points and covered a circular area of 1600 m^2^ (each) with a radius of 22.6 m on flat terrain. A total of 825 trees with a DBH ≥7 cm were present in the ten plots, including 66.2 % *F. sylvatica,* 16.5 % *C. betulus*, 10.4 % *Q. robur*, 2.7 % *A. pseudoplatanus*, 2.3 % *F. excelsior*, 1.5 % *L. decidua*, 0.2 % *P. avium*, 0.1 % *A. platanoides* and 0.1 % *B. pendula*. The stands of this study were dominated by deciduous tree species, and the forest complexity on the selected sample plots can be classified as ‘easy’ to ‘medium’ according to the classification of the TLS-based forest inventory benchmark study by Liang *et al.* (2018). The plots showed differences in tree species composition and tree density, the latter ranging from 211 to 997 trees ha^–1^. The characteristics of the plots are summarized in Appendix Table A1. To study the impact of scanning distance on tree detection and segmentation quality, we assigned the ten plots to three groups based on the distance of the plot centre from the MLS trajectory: 0–20 m (group A, *n* = 3), 20–40 m (group B, *n* = 4) and 40–60 m (group C, *n* = 3).

**Fig. 3.**
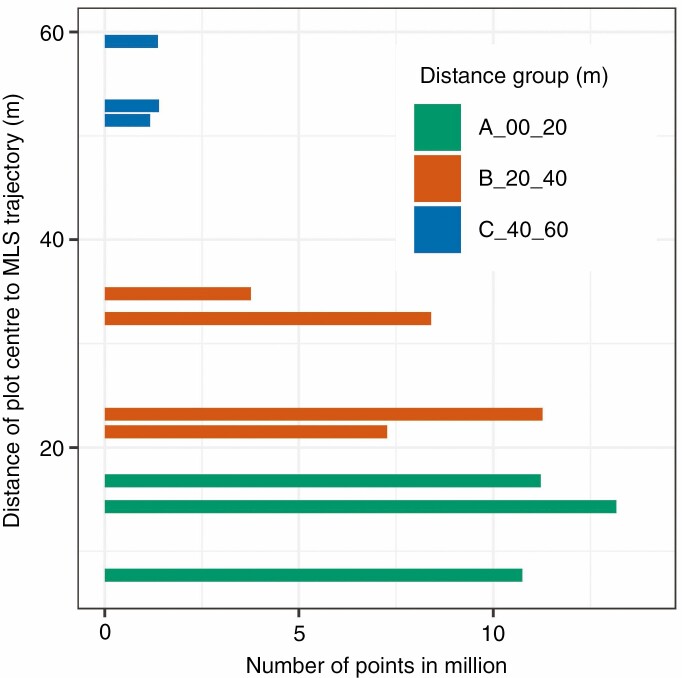
Grouped plots with number of points depending on the distance of the plot centre to the trajectory.

The MLS data from these plots were clipped to a vertical cylinder with a radius of 25 m to provide a buffer for crown segmentation. The forest track on which the MLS system was used had a distance *s* from 7 m ≤ *s* ≤ 59 m to the plot centres (Fig. 3).

### Conventional inventory

Measurement data from manual inventories (DBH and tree height) for the year 2013 were available for 74 trees within the sampling scheme of the inventory points. The DBH was measured with a caliper at 1.3 m height. Trees with a DBH <30 cm were measured once with an inward facing caliper and those with tree diameters >30 cm were measured twice (crosswise) and averaged. The tree height measurements were performed using conventional field measurements with a hypsometer.

For further analyses, three test areas (I, II and III) were selected from the MLS data of the study area. The areas were selected from a biodiversity perspective (plots with a mixture of different tree species and age classes) and they represent the total Lauerholz Forest: areas I and II display a high diversity of species and ages, and area III comprises mainly pure beech stands. In February 2019, another field campaign was carried out to perform a tree species identification in areas I, II and III. The tree locations and DBH were detected automatically in the MLS data. Within the MLS data, missing trees (without previous DBH measurement) were then added manually by marking them in the 2-D point cloud slice. All tree positions were stored as stem base maps right and left of the trajectory (depth of 35 m and length of 50 m along the trajectory). Based on these stem base maps, tree species identification was conducted *in situ* by two people over a period of 1 week. Trees that were present in the map but could not be found in the field campaign were identified, in contrast to the missing trees on the map, which were not further located and counted.

### Segmentation of MLS data

The MLS data are characterized by different point densities. The segmentation of forest MLS data is a challenge for any algorithm which deals with point distances, since the point density decreases with increasing distance from the trajectory. We have developed a distance-dependent algorithm that takes into account the inhomogeneities in point density and segments forest point clouds fully automatically.

Our algorithm requires point clouds as Cartesian *X*, *Y*, *Z* co-ordinates as well as the trajectory of the MLS journey with Cartesian *X* , *Y* and *Z* co-ordinates and a time stamp of the required points. The source code was written in C++ utilizing PCL 1.8.1 (Point Cloud Library – open source) (Rusu and Cousins, 2011). The result is the segmented point cloud, a parameter file (segment-wise tree parameters) and various 3-D data for visualization of the parameters [stem points, points of the DBH, profiles, crown projection area (CPA) and alpha shapes of the crown].

The algorithm consists of four processing steps which are carried out in one flow: (1) pre-processing, i.e. voxel space transformation of the data with digital terrain model (DTM) generation and ground elimination; (2) tree detection along horizontal sections (tree position = starting points of segmentation) based on the method of Maas *et al.* (2008); (3) iterative extension of the tree segments by distance and membership criteria; and (4) tree parameter determination.

#### (1) Pre-processing with voxel space transformation and DTM reduction.

**Fig. 4.**
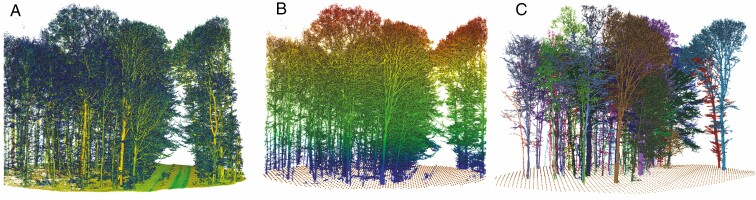
(A) MLS point cloud of plot A2 (colour-coded by intensity, plot radius 22.6 m); (B) filtered point cloud colour-coded by height without ground points and DTM points (brown, grid size 1 m); (C) segmented trees.

 In the first step, we transformed the irregular (unorganized) point cloud (Fig. 4A) into a voxel space using the voxel grid function of the PCL library. We chose a voxel size (*v*_size_) of 1 cm and only kept voxels that contain at least one point (*n* ≥1). The voxel space allows a fast and discrete addressing of voxels and their neighbours and is advantageous in terms of computing performance when compared with unorganized point clouds. After the voxelization, a DTM was derived with a defined grid spacing of 1 m (*s*_DTM_; Fig. 4B). The lowest *Z* co-ordinate of a voxel within a raster cell defined the height of the raster cell. Finally, the classified ground voxels were separated to exclude a connection of the trees over the ground. A pre-defined offset in the vertical axis (*ΔZ*) was used to filter the ground and understorey vegetation voxels grid by grid. All voxel cells within a DTM raster cell between the raster cell height *Z*_grid_ and *Z*_grid_ + *ΔZ* belonged to the class of ground voxels and were separated. The size of the offset depends on the height of the understorey vegetation and on the largest slope of the terrain that occurs in a raster in order to successfully separate the ground voxels. The terrain elimination step is essential for successful tree segmentation. In the further course of processing, the voxelized points were used and referred to as points. An accuracy of a decimetre at ground level was sufficient, as the ground points were primarily extracted to avoid a connection between the trees. There was a risk of trunks being cut off at the bottom, but the points were added back in the stem segmentation process step.

#### (2) Tree detection.

Tree trunk centres (i.e. tree position) at 1.30 m above the ground formed the basis for the seed points of the segmentation. To determine the tree positions, point clusters were detected and classified within three horizontal cuts above the DTM in the lower stem area (cut heights *h*_1_, *h*_2_, *h*_3_ to minimize occlusion). The three positions of the individual cuts were averaged and a provisional mean position was determined. The three slices were analysed by a 2-D quadratic structure element with a size *S*. In contrast to the tree detection approach applied for TLS data (Bienert *et al.*, 2007), a distance-dependent point number threshold *n*_min_ was used as the criterion for the point cluster search. The threshold was determined by the velocity of the platform, the shortest Euclidian distance of the point clusters to the MLS trajectory and the bounding box of the cluster. The bounding box was divided into equally spaced grids, and the maximum possible number of points *n*_max_ (clear view without occlusions on a vertical surface) was calculated for a grid element. The maximum number per bounding box resulted from the sum of all grids *g* occupied by points (*n*_box_ = g × *n*_max_). *n*_min_ resulted from 60 % of *n*_box_.

**Fig. 5.**
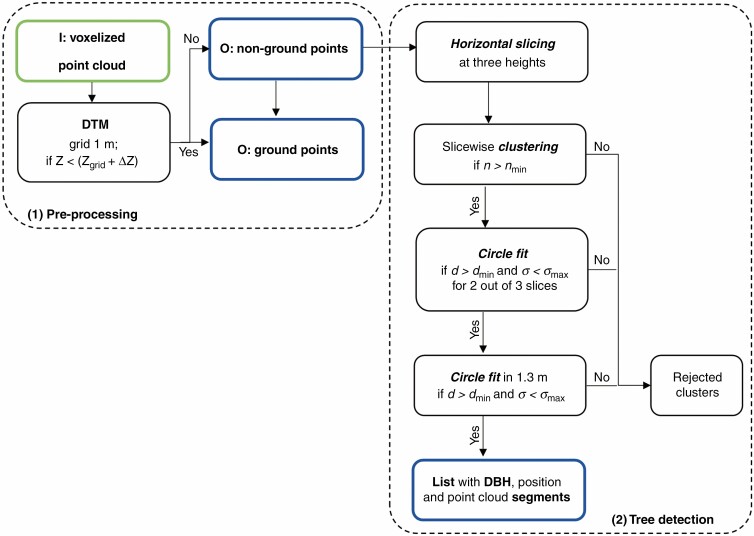
Flow chart of the processing steps 1 and 2: pre-processing and tree detection. The input data are outlined in green and the output of the step in blue.

If a point cluster had more than *n*_min_ points, a circle fit was started. If, in addition, in at least two out of three clusters of the three horizontal cuts the determined diameter *d* was >7 cm (*d*_min_) and the standard deviation of the circle fit (*σ*) was below a threshold value $\sigma_{\max}$, the object was considered to be a tree. With the mean stem diameter $\bar{d}$ (over the three diameters of the horizontal cuts) and the mean position of the tree detection and a buffer (+20 %), the stem points were selected at 1.3 m (±10 cm) above ground and defined the DBH segment. A circle fit with all points of the DBH segment determined the diameter *d*_1.3_. The centre point of the DBH defined the position of the tree. All potential tree candidates were collected in a list with their DBH segments and position, sorted by descending diameter *d*_1.3_. Figure 5 outlines the steps.

#### (3) Iterative extension of the stem and tree segments. a) Stem segmentation

Stem segmentation: based on the detected stem centre and the corresponding stem diameter, the stem points with outgoing branches were to be selected. First, the search radius *d*_T_ per tree T was defined by the DBH *d*_1.3_ and half of the distance to its nearest neighbour tree *d*_NN_ to avoid an oversegmentation [eqn (1)].


$$d_{T}=d_{1.3}+d_{NN}/2$$ (1)

The octree radius search function from the PCL library was then used to select contiguous points *P*_*i*_ which were less than a distance *r*_*T,i*_ from each other.


$$\begin{array}{l} r_{T,i}={\bar{d}}_{min}^{DBH}+\;d_{Z} \end{array}$$ (2)

The definition of *r*_*T,i*_ [eqn. (2)] results from a local mean point density of all minimum distances *d*_min*,i*_ of the DBH segment ${\bar{d}}_{min}^{DBH}$ a constant factor *f*_DBH_ [eqn. (3)] and a height-dependent distance *d*_*Z*_ [eqn (4)].


$$\begin{array}{l} {\bar{d}}_{min}^{DBH}=f_{DBH}*\frac{\overset{n}{\underset{i=0}{\sum }}d_{\min,i}}{n} \end{array}$$ (3)

$$\begin{array}{l} d_{Z}=|Z_{i}-Z_{tra}|\times f_{Z} \end{array}$$ (4)

*d*_*Z*_ results from the *Z* co-ordinate *Z*_*i*_ of the respective point *P*_*i*_, the Z co-ordinate *Z*_tra_ of the trajectory at the acquisition time of *P*_*i*_ and a constant height factor *f*_*Z*_. Starting from an arbitrary point of the DBH segment, the point spacing of the candidates was examined and any point whose spacing was smaller than *r*_*T,i*_ was added to the stem segment. Each assigned point was deleted from the non-ground point cloud and belongs to only one segment. In addition, the ground points were traversed with the same point spacing *r*_*T,i*_, except that the search was limited to a right circular cylinder with a radius of DBH/2 plus a buffer of 10 cm.

##### b) Crown segmentation

the crown segmentation process used the stem segment list from the previous processing step as input. First, the remaining non-ground points were clustered, and larger clusters were fragmented into sub-clusters with a size of 0.5 × 0.5 × 0.5 m. The pairwise distances were computed for each cluster (3-D centre of gravity) and stem segment. If the distance was within the search radius d_*T*_, the cluster was selected as a potential cluster candidate. The potential cluster candidates were sorted in ascending order of distance. The number of cluster candidates for each stem segment is n_max_.

In a second step, three nested iterations work into each other.

(i) Each stem segment *i* was examined with regard to the cluster with the shortest distance. If the cluster distance was the shortest in regard to the other stem segments and was smaller than a distance-dependent threshold *d*_*c*_ [eqn (5)], taking the tree distance *d*_*XY*_ with the 2-D distance to trajectory *d*_*2Dt*_ and a constant factor *f*_*XY*_ [eqn (6)] and the height *d*_*Z*_ [eqn (4)] of the cluster into account, the cluster was added to the stem segment. The cluster has been signed as allocated and was no longer taken into account in further counting. The next stem segment was examined until *i* = *n*.

$$\begin{array}{l} d_{c}=d_{XY}+d_{Z} \end{array}$$ (5)

$$\begin{array}{l} d_{XY}=d_{2Dt}\times f_{XY} \end{array}$$ (6)

1. Iteration (i) repeats until all clusters for each stem segment (with cluster number *n*_*max*_) in the search radius had been examined and either added to a stem segment or declared as unassignable.
2. The search radius was increased by Δ*d*_*T*_ and iteration (i) and (ii) started again. If a cluster was sufficiently close to multiple stems, the closest was chosen and was allocated to the stem segment. Each cluster could only be assigned once. The number of iterations depends on the size of Δ*d*_*T*_ and the number of potential clusters. As soon as no more potential clusters were found, the last iteration step was reached.

The outcome of the process is a stem segment list extended by branches, which is hereafter referred to as a tree segment list.

**Fig. 6.**
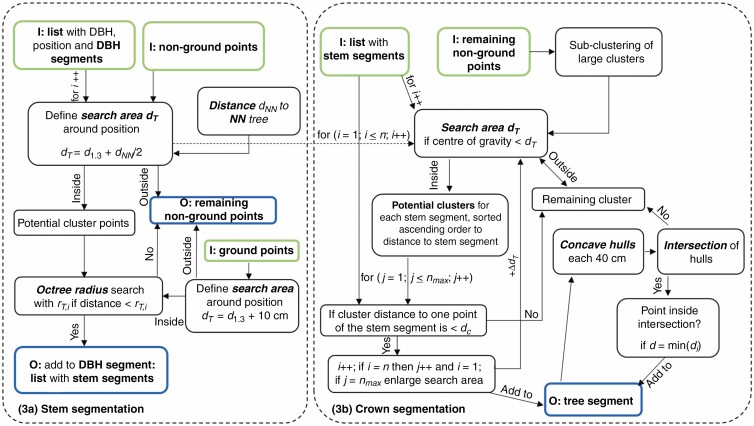
Flow chart of the processing step 3: stem segmentation and crown segmentation. The input data are outlined in green and the output of the step is in blue.

In the last step, each tree segment was divided into horizontal sections of equal heights (0.40 m) and the concave hulls were calculated section-wise. If no intersection with hulls of neighbouring trees was detected, the remaining points lying within the hull were assigned to the tree segment. If an intersection was registered, the points *i* within the common intersection were examined with respect to their distance to the points of a tree segment. The point was added to the tree segment (Fig. 4C) with the smallest distance. Figure 6 shows the steps for processing stem and crown segmentation.

#### (4) Tree parameter determination.

For each segmented tree object, tree-specific parameters were determined and stored. The derived parameters can be divided into five classes: location parameters, tree height and stem parameters, crown parameters and neighbourhood relationship parameters. Table 1 gives an overview of the parameters and their determination.

**Table 1.** Tree parameters determined during the segmentation algorithm.

	Name	Symbol	Unit	
**Location**	**Tree position**	*X*_t_	(m)	Co-ordinates (*X*, *Y*, *Z*) of the centre point of the DBH
	**Tree distance (2-D)**	*d*_2*Dt*_	(m)	Orthogonal distance from tree to trajectory
**Tree height**	**Tree height**	*h*_t_	(m)	Difference between tree top (highest) and foot (lowest) point
**Stem parameter**	**Diameter at breast height**	*d*_1.3_	(m)	Diameter at 1.3 m above terrain level with s.d. σ of the fitted diameter, diameter determination with a least-square circle fitting
	**Profiles with diameter and position**	$d_{pro}^{i\ldots n}$ $X_{pro}^{i\ldots n}$	(m) (m)	Diameter *d*_pro_ and position *X*_pro_ along the stem with constant interval and s.d. σ of the fitted diameter according to the DBH determination
	**Stem volume**	*V*_stem_	(m^3^)	Sum of all detected stem profile volumes (cylinder volume determined with the stem diameter and the constant interval as cylinder height)
**Crown parameter**	**Crown centre of gravity**	*X*_grav_	(m)	Co-ordinates (*X*, *Y*, *Z*) of the centre of gravity of all crown points per tree
	**Crown radius (min, max)**	$r_{C}^{min},\;r_{C}^{max\;\;\;}$	(m)	Maximum and minimum crown radius from centre of gravity (*XY* projection)
	**Crown width (min, max, mean)**	$cw^{min},\;cw^{max},\bar{cw}$	**(m)**	Widest crown width (max) and the resulting width perpendicular to this (min), mean width $\bar{cw}$ of min and max
	**Crown base height**	*h*_cb_	(m)	Height of first detected branch
	**Crown length**	*l*_C_	(m)	Difference between tree height *h*_t_ and crown base height *h*_cb_
	**Crown projected area**	CPA	(m^2^)	Crown projected area calculated with concave hull polygon CPA
	**Concave hull (per tree, slice wise)**	Hullconcave		Vector of polygon points (*X*,*Y*,*Z*) per tree and section-wise (section height 40 cm)
	**Live crown ratio**	LCR	(–)	Ratio of crown length to tree height
	**Crown index**	CI	(–)	Ratio of crown length to crown width (mean)
	**Alpha shapes**			Alpha shapes of the crown
**Neighbourhood relationship**	**Neighbouring trees (intersected crown)**	$n_{tree}^{inter}$		Vector of the number of neighbouring trees intersecting in the crown (concave hull)
	**Neighbouring trees (overshadow)**	$n_{tree}^{over}$		Vector of the number of neighbouring trees which completely overshadow each other

(a) *Location parameters:* tree position (co-ordinates of the circle centre of the DBH) and the orthogonal distance of the tree to the trajectory were determined for each tree.
(b) *Tree height:* the height was defined by the difference between the lowest and highest point of the segmented tree point cloud.
(c) *Stem parameters:* from the segmented tree point clouds, a set of stem parameters can be determined. Further diameters *d*_pro_ along the trunk were determined in constant sections starting from the position of the DBH (profile fitting). The total volume of the stem was derived from the sum of the cylinder volumes calculated from the diameters obtained by the profile fitting procedure. To detect under- and overestimated profile diameters, the profiles were run through with a median filter (5 × 1 kernel size) and smoothed if necessary. The diameter to be examined was replaced with the median value of the kernel when the difference between the median value and the diameter was >10 % of the diameter.
(d) *Crown parameters:* to determine the crown parameters, the tree was split into crown and lower trunk. This was done by identifying the crown base height (CBH; defined by the height of the first crown branch). From the separated crown points, the crown centre of gravity, the concave hull and the CPA based on the calculation of the concave hull were determined. Starting from the centre of gravity, the minimum and maximum crown radius of the 2-D projection was determined. The crown length *l*_C_ was derived from the crown base height *h*_cb_ and the tree height *h*_t_ [eqn (7)]. Additionally, the ratio of crown length to mean crown width $\bar{cw}$ [crown index CI; (eqn (8)] and the live crown ratio LCR [eqn (9)] per crown were stored. The LCR is the ratio of crown length to tree height and is an indicator for tree vigour.

$$\begin{array}{l} l_{c}=h_{t}-h_{cb} \end{array}$$ (7)

$$\begin{array}{l} CI\;=l_{c}\sqrt{\bar{cw}} \end{array}$$ (8)

$$\begin{array}{l} LCR\;=l_{c}/h_{t}\times \;100 \end{array}$$ (9)

**Fig. 7.**
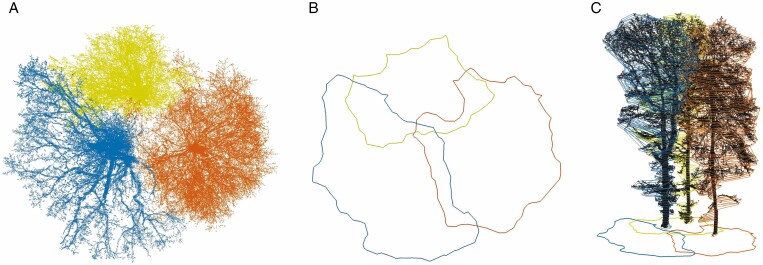
(A) Coloured MLS point clouds of a segmented tree group (plot A1) with corresponding crown polygons (B); (C) section-wise crown polygons (2.5-D). Yellow tree, *Q. robur* with a distance of 3.26 m from the trajectory; blue and orange tree: *F. sylvatica* with trajectory distances of 5.47 m and 10.43 m, respectively.

(e) *Neighbourhood relationship parameters:* the analysis of the crown polygons with respect to their position and common intersections with other crown polygons allowed the detection of the nearest tree neighbours that touch and influence each other in the crown area. The crown polygon of a tree was determined with a concave hull (using the ConcaveHull function of the PCL library) of all projected tree points (*Z* = 0). Moreover, in a 2.5-D approach, the program stored the crown polygons for the complete tree crown in 40 cm horizontal slices and detected the surrounding polygons along the crown (Fig. 7). A distinction was made between three cases, (1) A tree polygon *A* lay outside polygon *B* (there are no intersections *I* between the two treetop polygons, *I = *0). (2) A tree polygon *A* lay inside polygon *B* (thus, there are no intersections *I* between the two treetop polygons, *I = *0). Section-wise analysis of the 2.5-D polygons started with analysis of adjacent polygons (of the same heights) of neighbouring trees. If *I* ≥2, the tree crowns interacted with each other, otherwise the tree *A* was completely overtopped by treetop *B*. (3) In the 2-D projection of the polygons there were at least two intersections *I* ≥2. The trees were potential candidates whose treetops interact with each other. To what extent the crown spaces actually intersected was analysed by examining the crown polygons section-wise in 2.5-D.

### Segmentation of TLS data

Semi-automatically segmented TLS scans of plots acquired at the same time as the MLS data served as reference datasets for validation of the MLS segmentation. To provide accurate reference data, segmentation with manual intervention was unavoidable. All trees with a DBH ≥7 cm of the TLS plots were segmented in a stepwise procedure. First, an automatic segmentation was performed with the SimpleTree (4.33.06) plugin of the Computree (5.0.054b) software (Hackenberg *et al*., 2015*b*). SimpleTree is an open-source tool to create cylindrical tree models from terrestrial laser scanner point clouds with segmented point clouds as an intermediate result. In a second step, the segmented tree point clouds were visually checked, and incorrectly classified tree segments were manually eliminated or reallocated using the RIEGL RiSCAN Pro (2.6.1) software interface. In total, 825 trees were segmented with such a semi-automatic procedure in the ten sample plots, and each tree point cloud was stored in a separate file.

### Co-registration of MLS and TLS data

It was necessary to register the TLS data with the georeferenced MLS data because the position of the terrestrial laser scanner in the plot centre was not always exactly on the raster inventory point, and the GNSS accuracy of the TLS and MLS scanners’ own positioning system were insufficient due to the canopy cover (Bienert *et al*., 2018). Additionally, precise co-registration enabled data evaluation in a uniform co-ordinate system. For the transformation we used CloudCompare (2.6.3) software, and the TLS plots were iteratively registered with the MLS plots. The height offset to the MLS data was corrected manually by translating the TLS point cloud. A horizontal section was then cut out in both datasets using the segmentation tool. The horizontal layers were then roughly aligned by clicking on corresponding pairs of points [Registration tool: Align (point pairs picking)]. Finally, the point clouds were registered with the ICP (Besl and McKay, 1992) algorithm of the registration tool.

### Data analysis

#### (1) The analyses on the three sites.

**Fig. 8.**
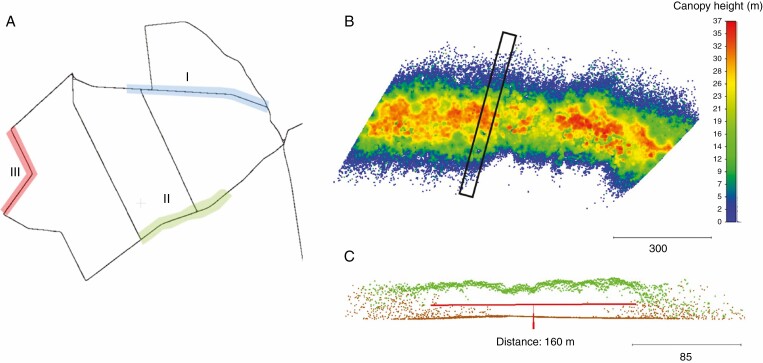
(A) Location of the test areas I, II and III in the study area Lauerholz Forest; (B) normalized digital canopy model (nDCM) of test area I; (C) side view of a point cloud section (30 m thickness, marked in A) of the DTM (brown) and nDCM (green) points.

 In the three test areas I, II and III (Fig. 8A), the following analyses based on MLS data were carried out:

(a) *DTM and DCM:* the DTM and digital canopy model (DCM) of the test areas were obtained with CloudCompare applying a grid size of 2 m. The normalized digital canopy model (nDCM – reduced canopy height by the height of the ground points) was visually analysed for test area I by plotting 50 m sections.
(b) *Tree detection:* for all trees within a 50 m transect to the right and left of the forest track (driving trajectory) of areas I, II and III, a tree detection was automatically performed (Maas *et al*., 2008) and the tree position and DBH were determined. Due to too few points on the trunk as a result of occlusions, some trees remained undetected; their position was added manually to the tree list for the tree detection analysis without DBH determination. Therefore, smaller stem diameters (DBH <7 cm) were also included. The manual interaction was only performed for the tree detection analysis.

#### (2) Comparison of MLS, TLS and conventional field measurements on the ten sample plots. (a) Segmentation: ....

(a) *Segmentation:* for the segmentation of the ten MLS plots, we used the proposed segmentation method with the proposed parameters and without manual interaction (see ‘Segmentation of MLS data’, and Table 2). The number of detected trees *n*_det_ within the plots was compared with the number of TLS trees, *n*_TLS_, from the segmentation, and the detection rate $p\%\;=n_{det}*\;100/n_{TLS}$ was calculated. The only difference between the TLS and MLS data processing was the way in which the trees were segmented (see ‘Segmentation of MLS data’).
(b) *Tree parameters:* in both datasets, the same algorithms were used to automatically determine the tree parameters. To validate the accuracy of the tree parameter extraction, the derived parameters (tree height, DBH and CPA) of the segmented MLS trees were compared with the manually measured inventory parameters (tree height and DBH) and with the extracted tree parameters of the TLS point cloud (tree height, DBH and CPA). The given differences Δ as well as the calculated RMSE refer to the reference values (inventory measurement or TLS) and were calculated with Δ = reference – MLS and Δ = reference – TLS. To validate the segmentation results, the segmented trees were transformed into a voxel space and the difference between TLS- and MLS-occupied voxels was calculated. The voxels that had no correspondence in both datasets represent an undersegmentation (remaining voxels in the TLS dataset) or an oversegmentation (remaining voxels in the MLS dataset) with respect to the MLS data. All statistical data analysis was performed with R (3.5.1; R Core Team, 2017).

**Table 2.** Parameters used for a full automatic processing chain of the plots.

Processing step	Parameter	Description
DTM generation and down sampling	*n* ≥1	• *n*: number of points inside a voxel cell
	*v*_size_ = 0.01 m	• *v*_size_: voxel size
	*s*_DTM_ = 1.00 m	• *s*_DTM_: DTM grid spacing
	ΔZ = 0.45 m	• ΔZ: offset of ground point classification
Tree detection	$\sigma_{max}$ = 0.05 m	• $\sigma_{\max}$: threshold for s.d. of the circle fit
	*S* = 0.10 m	
	1.3 m ≤ *h*_1_ ≤ 1.5 m	• *S*: size of structure element for cluster analysis
	2.0 m ≤ *h*_2_ ≤ 2.2 m	
	2.9 m ≤ *h*_3_ ≤ 3.1 m	• *h*_1_, *h*_2_*, h*_3_: cut heights for tree detection
Stem segment and tree segments	$\Delta d_{T}$ = 1.5 m	• $\Delta d_{T}$: delta for increasing the search area
	*f*_DBH_ = 1.2	• *f*: constant factor for distance-dependent threshold
	*f*_*XY*_ = 0.002	
	*f*_*Z*_ = 0.0015	

## Results

### The analyses on the three test sites

#### (1) MLS data quality.

**Fig. 9.**
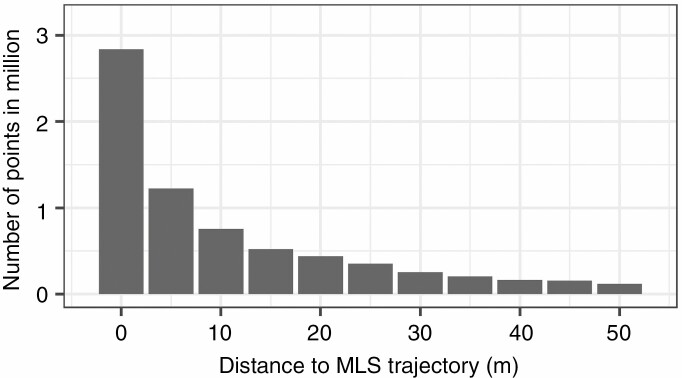
Histogram with the number of MLS points per parallel section (averaged over a length of 80 m).

Figure 9 presents a histogram with the number of MLS points per parallel section to the trajectory over a length of 80 m. The nearest-neighbour distance decreases with increasing distance from the trajectory and with increasing tree height. The histogram shows the combined effects of occlusions and a decrease in point density.

#### (2) DTM and DCM.

The visual analysis (Fig. 8C) of the normalized DCM (Fig. 8B) shows errors as a result of scan occlusions in the DTM and DCM, which increase from 80 m to the left and right of the forest track. An analysis of the laser point distribution shows that the scan points reflect the mean stand height up to a distance of about ±50 m from the trajectory. At distances greater than ±50 m, the stand height decreases due to insufficient sampling in the crown (occlusions).

#### (3) Tree detection.

A total of 6316 tree positions were detected automatically and 2251 trees were interactively detected (trees with DBH <7 cm included). Finally, the 8567 obtained tree positions were saved as stem base maps and were checked manually in a field campaign. Overall, 26 % of the 8567 trees were not detected automatically, including trees which were severely occluded and trees with a DBH <7 cm, which was an exclusion criterion in the detection algorithm from the outset. Appendix Fig. A1 shows the number of trees that were subjected to fully automatic MLS tree detection and manual added MLS trees across test sites I, II and III. The first bin contains only a few trees, which is due to the fact that the forest track from which the scans were made had a width of 5–8 m in parts and thus only a few trees were located at the edge within the bin. Furthermore, Appendix Fig. A1 shows a clear decrease in the number of trees detected when assuming a homogeneous stand distribution, be it automatically or manually added trees. This indicates occlusions in terms of stand density and decreasing MLS scan point spacing. Thus, the number of detected trees along the trajectory is affected by the distance to the trajectory. It should be mentioned again that the manually added trees were based on the MLS point cloud without DBH determination. No statement can be made about the number of true trees (or missing trees), as only the number of manually added trees was known and evaluated.

### Comparison of MLS, TLS and conventional field measurements on the ten sample plots

#### (1) Tree detection and segmentation within the plots.

The automatic detection rate for trees with DBH ≥7 cm was in the order of >94 % for about half of the plots (Table 3), but significantly dropped in difficult cases with dense understorey vegetation. The rate depends strongly on the distance to the trajectory and the plot density (shading due to tree density and understorey vegetation). Two plots had an unsatisfactory detection rate of 10.3 % and 22.5 %. These plots had a large distance to the trajectory (51.5 m and 59.1 m) and were young beech (*F. sylvatica*) stands with dense understorey. The analysis of the subtracted voxel spaces with a voxel size of 40 cm showed an average undersegmentation of 61 % and an oversegmentation of 16 %. Appendix Table A2 presents the percentage rates of over- and undersegmentation per plot. Due to the varying distance of the plot centres to the trajectory and the tree counts, the point count of the MLS data varies and adopts values from 1.3 million to 13.3 million points. The fully automated process of tree detection with final segmentation took between 9 and 123 min, depending on the plot characteristics (Intel® Core i7-6700, 2.6 GHz, 16 GByte RAM, 64 bit).

**Table 3.** Characteristics and detection rate for automatic tree detection of the reference forest plots

	Plot name	Distance from the plot centre to the trajectory (m)	No. of MLS points in Mio	Plot description	MLS-detected trees	TLS-detected trees	Detection rate (%)	Segmentation time (min)
**A**	**1**	7.7	10.7	Dense understorey vegetation, forest track included	33	34	97.1	90
	**2**	14.3	13.1	Young stock, forest track included	97	100	97.0	112
	**3**	16.8	11.2	Forest track, young stock with deep-seated branches	47	50	94.0	123
**B**	**1**	21.5	7.2	Dense understorey vegetation, ditch	37	46	80.4	75
	**2**	23.2	11.2	Forest track, sparse understorey vegetation	79	82	96.3	85
	**3**	32.4	8.4	Lying trees, ditch	107	141	75.9	45
	**4**	34.8	3.7	Young stock with understorey vegetation	37	42	88.1	100
**C**	**1**	51.5	1.2	Dense stand with many young trees	11	107	10.3	9
	**2**	52.9	1.4	Open plot, no understorey vegetation	58	63	92.1	20
	**3**	59.1	1.3	Dense stand with many young trees	36	160	22.5	11

The MLS trees were detected and segmented automatically and the TLS trees were detected and segmented semi-manually.

**Fig. 10.**
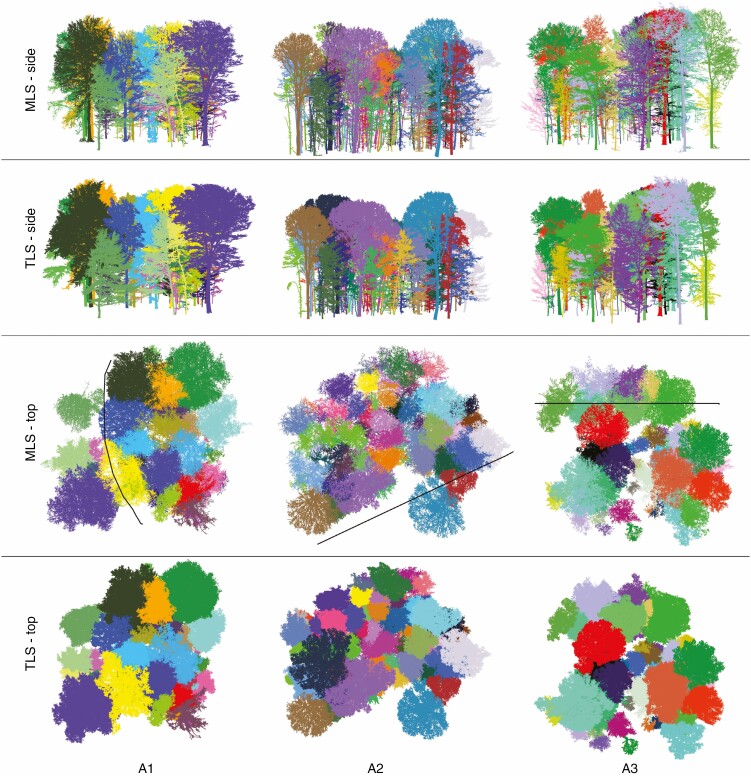
Automatically segmented MLS plots compared with the semi-automatically segmented TLS plots (black line shows the MLS trajectory). Individual trees are indicated by different colours and only the corresponding trees are displayed. For printing purposes, the same visualization parameters were used for both datasets.

**Fig. 11.**
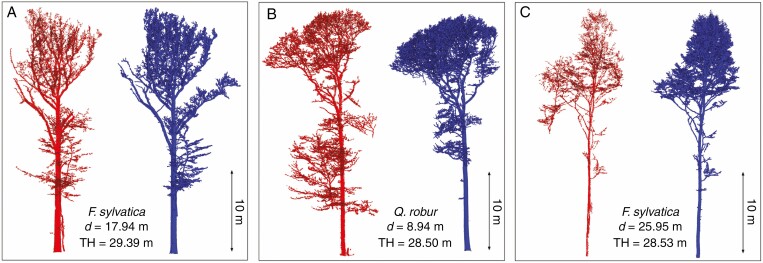
Examples for undersegmented (A) and oversegmented (B, C) MLS trees (red) in comparison with TLS trees (blue) with distances to trajectory (*d*) and tree height (TH). The segmented TLS tree acts as a reference, and missing tree parts in the MLS data are considered as undersegmentation, and obviously additional branches (from neighbouring trees) are considered as oversegmentation. Trees from two plots of group A (A1 and A2). For printing purposes, the same visualization parameters were used for both datasets.

**Fig. 12.**
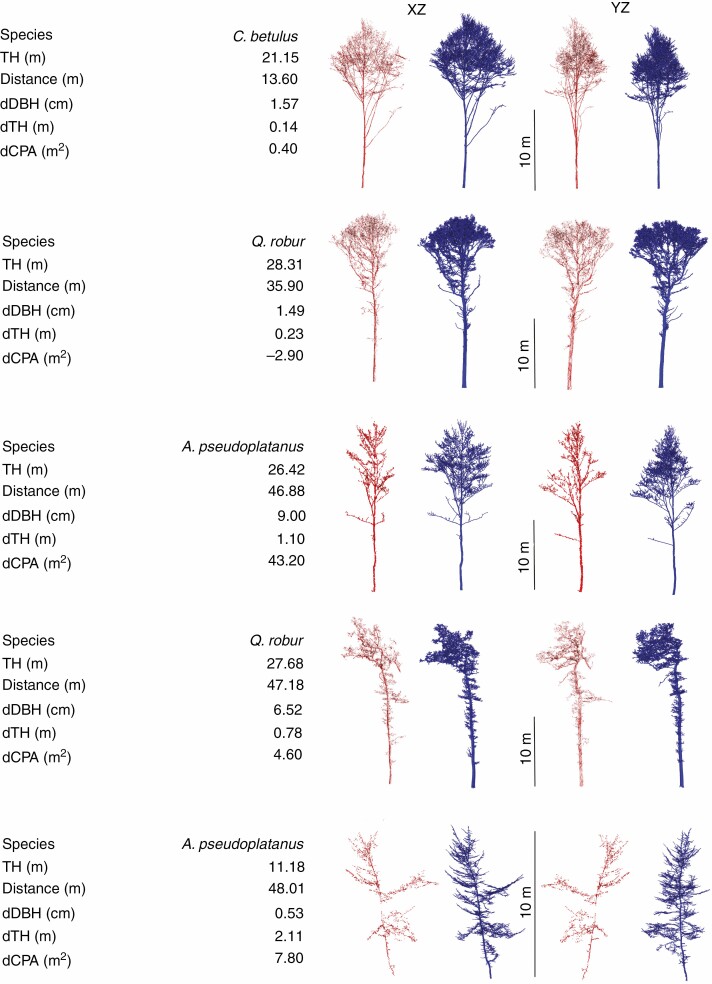
Segmented leaf-off tree point clouds in *XZ* and *YZ* projection: red, MLS data; blue, TLS data. The MLS point cloud was segmented automatically and the TLS tree point cloud was segmented semi-manually to serve as a reference [Δ = TLS – MLS; results from plot group B (B3 and B4)]. For printing purposes, the same visualization parameters were used for both datasets.

In Figure 10, the segmented MLS point clouds are compared with the results of the segmented TLS trees for group A (distance to plot centre <20 m). The segmentation results for groups B and C are given in Appendix Figs A2 and A3). The results show that the crown boundaries are visually comparable with the TLS trees in group A and in most cases also for group B, but in group C the crown segmentation is not satisfactory. Figure 11 presents three example trees for under- and oversegmented MLS trees in comparison with semi-automatically segmented TLS trees with different distances to the trajectory. The quality of fully segmented branches depends on the point cloud density. Despite distance-dependent segmentation, over- or undersegmentation occurred in places where the scan occlusions were too large or where branches from neighbouring trees touched. Figure 12 shows individual examples of automatically segmented MLS data (without manual correction) with different tree height, species and distances to the trajectory compared with segmented TLS data. Depending on stand density, even trees up to 48 m from the trajectory (one-sided scan) were segmented, with some gaps (Fig. 12). Especially at long distances, an increase in scan shading was observed; this is associated with gaps in the trunk and crown area. Nevertheless, it was possible to bridge these gaps, and the tree was recognized as such and some of the associated branches could be segmented.

#### (2) Tree parameters.

**Fig. 13.**
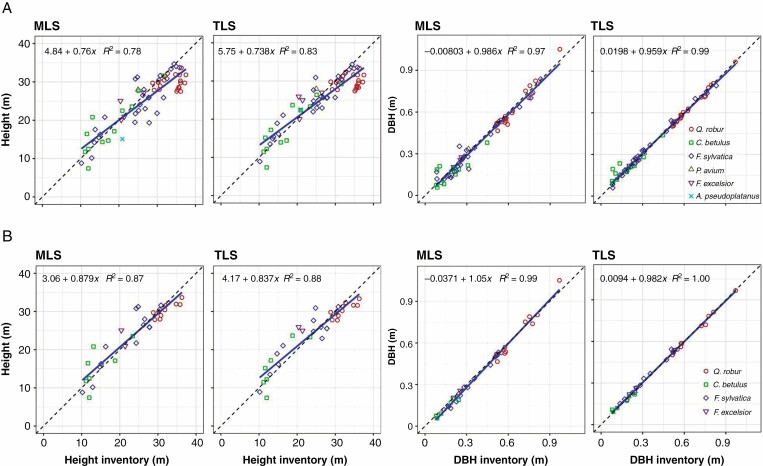
Tree height and diameter at breast height (DBH) from conventional inventory compared with MLS and TLS data-derived parameters of 74 trees on ten sample plots (A) and 40 trees including the plots with a tree detection rate >94 % (B).

Compared with the conventionally determined tree parameters tree height (TH) and DBH, we found a strong (TH) and a very strong (DBH) correlation (*R*_TH_^2^ = 0.78 for MLS and *R*_TH_^2^ = 0.83 for TLS, and *R*_DBH_^2^ = 0.97 for MLS and *R*_DBH_^2^ = 0.99 for TLS, respectively; Fig. 13A; Table 4) for all trees (*n* = 74). Considering the trees (tree count *n* = 40) from the four nearest plots with a tree detection rate >94 % (Table 3), we found a higher correlation (*R*_TH_^2^ = 0.87 for MLS and *R*_TH_^2^ = 0.88 for TLS, and *R*_DBH_^2^ = 0.99 for MLS and *R*_DBH_^2^ = 1.00 for TLS, respectively; Fig. 13B). The comparison produced an RMSE of the differences (Δ_man-laser_) of RMSE_TH_MLS_ = 4.22 m and RMSE_TH_TLS_ =  3.58 m, and RMSE_DBH_MLS_ = 45 mm and RMSE_DBH_TLS_ = 24 mm for all trees (Table 4). As expected, the selected trees (tree detection rate >94 % and plot distance of <24 m) delivered better RMSE (RMSE_TH_MLS_ = 2.96 m and RMSE_TH_TLS_ = 2.87 m, and RMSE_DBH_MLS_ = 32 mm and RMSE_DBH_TLS_ = 14 mm).

**Table 4.** RMSE and coefficient of determination (*R*^2^) of the TLS and MLS parameters tree height (TH) and diameter at breast height (DBH) compared with the manually determined values, divided by group

	Group		A (*n* = 27)	B (*n* = 35)	C (*n* = 12)	>94 % (*n* = 40)	All (*n* = 74)
	Plot distance to trajectory (m)		0–20	20–40	40–60	0–23	0–60
**TLS vs. manual**	TH	*R* ^2^	**0.90**	0.79	0.79	0.88	0.83
		RMSE (m)	**2.87**	4.03	3.88	**2.87**	3.58
	DBH	*R* ^2^	**1.00**	0.98	**1.00**	1.00	0.99
		RMSE (mm)	**14**	31	16	**14**	24
**MLS vs. manual**	TH	*R* ^2^	0.86	0.81	**0.87**	0.87	0.78
		RMSE (m)	3.12	4.11	6.24	**2.96**	4.22
	DBH	*R* ^2^	**0.99**	0.96	0.97	0.99	0.97
		RMSE (mm)	34	48	54	**32**	45

The highest values are highlighted in bold.

Growth increment could play a role in the differences found between the values measured in the field (2013) and the scanned data (2017). Both scan datasets (TLS and MLS) show similar magnitudes in the deviation of the tree height determination. However, when looking at the tree height determination results in detail (Fig. 13A), both positive and negative deviations are visible, which would appear to indicate a difficulty in defining the tree top during the manual inventory.

The comparison with the conventional inventory data shows that tree height from MLS data was determined with inconsistent accuracy over the different distances (RMSE values from 3.12 m up to 6.24 m; Table 4). Nevertheless, the smallest RMSE is at a height of RMSE_TH_MLS_ = 3.12 m for MLS and RMSE_TH_TLS_ = 2.87 m for TLS. DBH and tree height were determined with the greatest accuracy for the trees from plots closest to the MLS trajectory, as shown in Table 4.

**Fig. 14.**
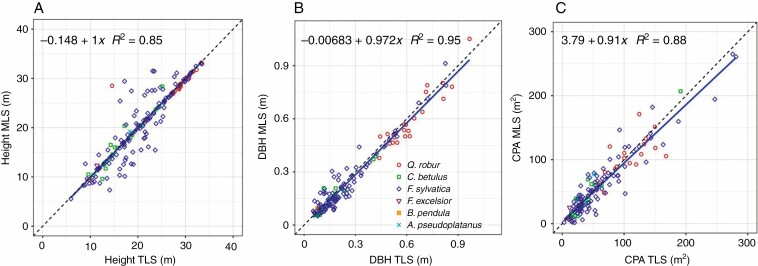
MLS and TLS tree height (A), DBH (B) and CPA (C) determined for all trees in group A.

The comparison in Table 5 and Fig. 14 of RMSE, mean difference and the coefficient of determination of the derived tree parameters (tree height, DBH and CPA) from TLS and MLS data focuses on the three main tree species (*F. sylvatica*, *C. betulus* and *Q. robur*) most commonly found in group A. Tree height was determined with the greatest accuracy for *Q. robur*, whereas CPA and DBH were most accurately determined in *C. betulus*. Overall, tree height and DBH based on MLS data are underestimated (${\bar{\Delta }}_{TH}$ = 0.23 m, ${\bar{\Delta }}_{DBH}$ *= 14.5 mm*), whereas CPA shows an overestimation (${\bar{\Delta }}_{CPA}$ = –5.61 m^2^). The difference between the tree species may be due to the architecture of the tree species (branch and crown structure) or the distance of the respective tree species to the trajectory. However, the distances were not investigated for the individual tree species.

**Table 5.** RMSE, mean difference $\left(\bar{\Delta }\right)$ and coefficient of determination (*R*^2^) of the MLS data-derived parameters tree height (TH), diameter at breast height (DBH) and crown projection area (CPA) compared with the TLS data-derived parameters for the three main tree species of group A (TLS – MLS)

		*F. sylvatica*	*C. betulus*	*Q. robur*	All three main species
	*n*	105	15	23	143
**TH** **(m)**	*R* ^2^	0.81	0.93	**0.99**	0.95
	RMSE	3.10	**1.42**	2.75	2.91
	$\bar{\Delta }$	0.32	–0.32	**0.28**	0.23
**DBH (mm)**	*R* ^2^	**0.92**	0.90	0.88	0.84
	RMSE	47	**36**	61	48
	$\bar{\Delta }$	**12**	14	30	15
**CPA** **(m** ^**2**^)	*R* ^2^	0.89	**0.95**	0.43	0.81
	RMSE	17.69	**11.58**	25.47	18.37
	$\bar{\Delta }$	**1.61**	–12.97	–31.55	–5.61

The highest values are highlighted in bold.

## DISCUSSION

Our analyses of the entire study area showed that the canopy heights and tree positions can be derived for >50 % of the trees up to a distance of 30 m to the right and left of the forest track. From this, in turn, it was possible to determine the forest compartment boundaries in terms of stem number and height distribution. Both canopy height (vertical component) and the distance from the trajectory (horizontal component) have a significant effect on the point spacing between neighbouring scan points at increased distances. With increasing distance of the reflected laser pulse from the trajectory, the mean point distance of the nearest neighbours increases. This effect is enhanced in the forest canopy and with dense ground vegetation due to shading effects.

The algorithm presented here is independent of the MLS system and works with a point cloud (*X*, *Y*, *Z*) and a trajectory in format (time, *X*, *Y*, *Z*). Thus, in theory, any LiDAR system with a trajectory-based terrestrial scan can be used. Since the segmentation algorithm has no restrictions regarding tree size, even smaller objects (understorey vegetation) were detected and segmented. Hence, the number of segments obtained is higher than the number of actual trees in the stand. However, by setting a threshold for the DBH, trees (or even other objects) with certain diameter classes can be distinguished. Since our study areas were located in a natural forest, a few man-made objects (trail markers, high seats) or even walkers were included in the scan data. These in turn can appear as potential tree candidates in the tree detection if the point arrangement is unfavourable. By a linked query of tree parameters, such as the tree height in combination with the stem profiles and the CPA, untypical tree objects can be eliminated at a later point.

Our sample plots were located in a mixed forest with a wide range of tree species, stand ages and tree counts. The results of the tree detection show that under the conditions of one-sided scan recording, the different tree counts and plot distances up to 23 m, 94–97 % of the trees (DBH >7 cm) are consistently detected (that means plots A1, A2, A3 and B2). With increasing distance to the trajectory, the detection rate decreases. Another factor influencing the quantity of detection and even the quality of segmentation is the one-sided acquisition of MLS data from a forest track, whereas TLS data are often acquired with 360° coverage. During MLS data acquisition along forest tracks, occlusions are caused by track-bounding understorey trees and herbaceous vegetation.

In order to increase the tree detection rate, cluster analysis should be carried out in several height sections, as the stems become visible at different heights due to occlusions by branches or smaller trees. In dense stands, where the lower stem section is completely hidden, the algorithm presented here would segment the trees only from the detection height. However, in most cases, only partial occlusions of the stems occur, and these can be successfully bridged by the search radius during stem segmentation. In our study, we performed cluster analysis at three point cloud heights.

The RMSE in tree height shows similar values for MLS (2.96 m) and TLS (2.87 m) compared with the conventional inventory data of 40 selected trees from the ≥94 % detection rate sub-group. This may also be partly attributed to the different year of data collection (2013 vs. 2017), since no correction was done, as well as to the inaccuracy of manual tree height measurement in dense stands. The RMSE of the MLS DBH is 32 mm and thus twice as large as the RMSE of the TLS. The reasons for this are the one-sided scan coverage, the lower scan point density and the accuracy of the MLS data registration.

The DBH and tree height obtained from MLS data compared with TLS-derived parameters show over- and underestimations scattered around the TLS value, but with a tendency toward underestimation (Table 5). The occlusion in the crown area with increasing distance leads to smaller detected tree heights, as the highest tree parts of distant trees are not fully captured.

The position of the tree parameters and the DBH remain largely unaffected by the quality of the segmentation. For example, poor canopy segmentation can still result in accurate tree parameters, such as DBH and position, as long as the stem sections have been segmented. The quality of the segmentation influences tree height determination of understorey trees; if the delineation of the crown is inaccurate, the determined tree heights are too large. The determination of these tree parameters is somewhat limited by registration inaccuracies ($\sigma_{mean}$ = 9 mm), insufficient point density and the scan coverage. The DBH and tree height are determined more accurately from the TLS point clouds. Crown parameters for which outer crown margin is taken into account (such as CPA, crown width and crown radius) are significantly influenced by the segmentation result. Despite shading in the canopy, the CPA is over- and underestimated from the MLS data with a tendency to slight undersegmentation, caused by the segmentation method in connection with the concave hull of the CPA calculation (Fig. 14). An undersegmented tree crown results in an oversegmented neighbouring crown. The visual comparison of the silhouette nevertheless showed good similarities to the TLS data (Fig. 10). The undersegmentation increases with increasing distance to the trajectory, which can be explained by the occlusions and the lower point density. In general, the undersegmented areas are mainly located in the interior of the crown. Another reason why the undersegmentation is larger than the oversegmentation is the one-sided stem recording, whereas the TLS reference data show a 360° coverage of the tree.

The advantage of MLS compared with TLS is the fast corridor-wide forest inventory. Compared with TLS (with several optimally arranged points of view), the lateral recording with the MLS results in more occlusions, which in turn can be minimized by multiple drives. This is due to the inclined arrangement of the two laser scanners of the Mobile Mapping System, which have a different angle of view of the forest depending on the direction of travel, thus eliminating some occlusions. The use of distance- and height-dependent segmentation radii delivers usable segmentation results up to a distance of 40 m (Appendix Fig. A2). Occasionally, very good segmentation results, visually compared with the TLS segmented trees, were also shown at distances of up to 48 m in plot B4 (tree density 262 trees ha^–1^; $\bar{d_{1.3}}$ = 38 cm). In general, the segmentation accuracy decreases from a distance of 40 m, with some exceptions. A clear advantage of automatic segmentation routines is the aspect of time, which is significantly reduced compared with time-consuming manual segmentation.

A disadvantage of the presented segmentation algorithm is that undetected trees at the beginning of the process chain are not considered in the further course. In the worst case, these tree parts (branches and twigs) are assigned to other tree segments when touching or falling below the search distance. For example, if a branch section was wrongly assigned, this section is missing for another tree because the points can only be assigned to one tree segment. This incorrect assignment, especially at the crown margins, leads to inaccuracies in the determination of crown parameters.

The application to pure coniferous stands has not yet been investigated. However, it can be expected that if there is no strong interaction (branch contact) between the trees, the segmentation algorithm may also be successfully applied, even when dealing with intermixed coniferous stands. In this study, the RIEGL VMX-250 Mobile Mapping System with a scan rate of 300 kHz and a speed of 10–15 km h^–1^ was used. As long as a registered point cloud with sufficient ground points is given, the algorithm can be applied to it. Grid-like tree arrangements (as often found in planted forest plots) could lead to shading of complete tree trunks, since MLS only detects trees from one side. Here, a meander-shaped trajectory (within the plot) would be recommended to minimize the occlusion in the trunk area. For the processing of TLS data (without trajectory information) the input parameters have to be redefined, since the density of co-registered multiple scans does not depend on distance to the scanner. The MLS data examined here show a straight-line course of the trajectory. As soon as major changes in the direction occur, e.g. due to track crossings, the trees in the crossing area are recorded at different distances, which leads to large differences in the determination of the distance-dependent segmentation radius. In these cases, the algorithm reaches its limits. One possible way to bridge the gap in the sometimes enormous differences in distance for the calculation of the distance-dependent threshold would be to determine the local point cloud density without taking the distance into account, e.g. by a NN distance.

Our study was able to show that scan distance affects the segmentation, but only beyond a certain distance (40 m). Beyond a distance of 40 m from the trajectory, the decreased point density means that even a trained manual segmentation operator reaches their limits in the crown area. By applying the distance-dependent segmentation algorithm, inhomogeneous point cloud densities can be processed. However, MLS data scanned on one side (as in this study from a forest track) show scan shadows from a certain distance onwards, as the algorithm is then stretched to its limits. Personal laser scan (PLS) data can be a good alternative with which to close the gap of the scan coverage. The accuracy of tree height determination is usually poor (Chen *et al*., 2019). Alternatively, however, tree heights can be determined very easily and with much higher accuracy from area-wide ALS scans (Y. Wang *et al*., 2019*b*).

### CONCLUSIONS

Here we present an algorithm for performing a tree-wise segmentation of MLS data of forest stands. Additionally, inventory relevant stem and crown parameters were determined for each detected tree. The algorithm was tested and validated on MLS data for a long forest corridor that included ten reference sample plots with a total area of 1.6 ha, 825 segmented trees and distances up to 60 m to the trajectory.

The algorithm presented here enables repeated, non-destructive and large-scale (corridor-wide) monitoring of forests at high spatial resolutions in the millimetre to centimetre range. By segmenting the individual trees on the basis of 3-D point clouds, manifold individual tree parameters – beyond DBH and height – can be derived in a very short time compared with conventional manual inventory. This enables new insights and opportunities for the generation and study of tree growth and interactions, e.g. competition and facilitation, and their underlying mechanisms over time.

The presented algorithm reveals several difficulties. First, the segmentation quality and the tree detection rate decrease with distance despite distance-dependent thresholds. The results showed a detection rate of trees with a DBH ≥7 cm up to 97 % for the very closest plots (<20 m) and an accurate crown delineation for the analysis of neighbourhood interactions. In the 20–40 m section, the tree detection rate reached values from 75 % up to 96 %. The most distant plots (>40 m) showed partially poor detection rates of 10–22 %. For one plot, we achieved a detection rate of 92 %, due to the stand and the sparse ground vegetation. With increasing distance from the trajectory, the decreased point density, together with increased occlusions, affects the quality of the segmentation result and in turn also influences the accuracy of the crown parameter determination. In our data, reasonable results could be obtained up to a distance of approx. 30 m to the MLS vehicle track. According to Appendix Fig. A1, the number of manually added trees (undetected) is predominant from 30 m upwards. Furthermore, track crossings, where trees are detected from different distances, represent a special case in the determination of the distance-dependent parameters, which has not yet been taken into account. A possible approach towards closing this gap could be the use of a local point cloud density.

Finally, the algorithm should be further developed, especially with respect to processing time and the segmentation of coniferous stands and urban roadside trees, with research into the latter already in progress. Overall, compared with plot-wise TLS data, forest MLS data allow for corridor-wise terrestrial data acquisition and are thus well suited to close any gap between plot-wise terrestrial data and area-wide ALS data.

## SUPPLEMENTARY DATA

Supplementary data are available online at https://academic.oup.com/aob and consist of the following. Table A1: characteristics of ten sample plots sorted by distance from the MLS trajectory. Table A2: percentage rates of the over- and undersegmentation of all plots. Figure A1: number of automatically detected MLS trees and undetected manually added trees based on MLS data divided according to distance from the trajectory. Figure A2: automatically segmented MLS plots compared with the semi-automatically segmented TLS plots of group B. Figure A3: automatically segmented MLS plots compared with the semi-automatically segmented TLS plots of group C.

mcab087_suppl_Supplementary_Tables

mcab087_suppl_Supplementary_Figure_S1

mcab087_suppl_Supplementary_Figure_S2

mcab087_suppl_Supplementary_Figure_S3

## References

[CIT0001] Aschoff T , Spiecker H. 2003. Algorithms for the automatic detection of trees. International Archives of Photogrammetry, Remote Sensing and Spatial Information Sciences 36: 71–75.

[CIT0002] Aschoff T , Thies M, Winterhalder D, Kretschmer U, Spiecker H. 2004. Automatisierte Ableitung von forstlichen Inventurparametern aus terrestrischen Laserscannerdaten. Wissenschaftlich-Technische Jahrestagung der Deutschen Gesellschaft für Photogrammetrie, Fernerkundung und Geoinformation 15: 341–348.

[CIT0003] Bauwens S , Bartholomeus H, Calders K, Lejeune P. 2016. Forest inventory with terrestrial LiDAR: a comparison of static and hand-held mobile laser scanning. Forests 7: 127.

[CIT0004] Besl PJ , McKay ND. 1992. A method for registration of 3-D shapes. IEEE Transactions on Pattern Analysis and Machine Intelligence 14: 239–256.

[CIT0005] Bienert A , Scheller S, Keane E, Mullooly G, Mohan F. 2006. Application of terrestrial laser scanners for the determination of forest inventory parameters. International Archives of Photogrammetry, Remote Sensing and Spatial Information Science 36.

[CIT0006] Bienert A , Scheller S, Keane E, Mohan F, Nugent C. 2007. Tree detection and diameter estimations by analysis of forest terrestrial laserscanner point clouds. ISPRS Workshop on Laser Scanning 2007 and SilviLaser 2007, 12–14 September 2007, Espoo, Finland, 50–55.

[CIT0007] Bienert A , Queck R, Schmidt A, Bernhofer C, Maas HG. 2010. Voxel space analysis of terrestrial laser scans in forests for wind field modelling. International Archives of Photogrammetry, Remote Sensing and Spatial Information Science 38: 92–98.

[CIT0008] Bienert A , Georgi L, Kunz M, Maas HG, von O heimb G. 2018. Comparison and combination of mobile and terrestrial laser scanning for natural forest inventories. Forests 9: 395.

[CIT0009] Burt A , Disney M, Calders K. 2019. Extracting individual trees from lidar point clouds using treeseg. Methods in Ecology and Evolution 10: 438–445.

[CIT0010] Cabo C , Del Pozo S, Rodríguez-Gonzálvez P, Ordóñez C, González-Aguilera D. 2018. Comparing terrestrial laser scanning (TLS) and wearable laser scanning (WLS) for individual tree modeling at plot level. Remote Sensing 10: 540.

[CIT0011] Calders K , Newnham G, Burt A, et al. 2015. Nondestructive estimates of above-ground biomass using terrestrial laser scanning. Methods in Ecology and Evolution 6: 198–208.

[CIT0012] Čerňava J , Tuček J, Koreň M, Mokroš M. 2017. Estimation of diameter at breast height from mobile laser scanning data collected under a heavy forest canopy. Journal of Forest Science 63: 433–441.

[CIT0013] Chen S , Liu H, Feng Z, Shen C, Chen P. 2019. Applicability of personal laser scanning in forestry inventory. PLoS One 14: e0211392. 30811414 10.1371/journal.pone.0211392 PMC6392439

[CIT0014] FAO . 2020. Global forest resources assessment 2020: main report. Rome: FAO. http://www.fao.org/documents/card/en/c/ca9825en/

[CIT0015] FAO and UNEP . 2020. The state of the world’s forests 2020. Forests, biodiversity and people. Rome: FAO. 10.4060/ca8642en.

[CIT0016] Georgi L , Kunz M, Fichtner A, et al. 2018. Long-term abandonment of forest management has a strong impact on tree morphology and wood volume allocation pattern of European Beech (*Fagus sylvatica* L.). Forests 9: 704.

[CIT0017] Hackenberg J , Wassenberg M, Spiecker H, Sun D. 2015 *a*. Non destructive method for biomass prediction combining TLS derived tree volume and wood density. Forests 6: 1274–1300.

[CIT0018] Hackenberg J , Spiecker H, Calders K, Disney M, Raumonen P. 2015 *b*. SimpleTree – an efficient open source tool to build tree models from TLS clouds. Forests 6: 4245–4294.

[CIT0019] Henning JG , Radtke PJ. 2006. Ground-based laser imaging for assessing three-dimensional forest canopy structure. Photogrammetric Engineering & Remote Sensing 72: 1349–1358.

[CIT0020] Jaakkola A , Hyyppä J, Kukko A, et al. 2010. A low-cost multi-sensoral mobile mapping system and its feasibility for tree measurements. ISPRS Journal of Photogrammetry and Remote Sensing 65: 514–522.

[CIT0021] Kukko A , Kaartinen H, Hyyppä J, Chen Y. 2012. Multiplatform mobile laser scanning: usability and performance. Sensors 12: 11712–11733.

[CIT0022] Kunz M , Fichtner A, Härdtle W, Raumonen P, Bruelheide H, von O heimb G. 2019. Neighbour species richness and local structural variability modulate aboveground allocation patterns and crown morphology of individual trees. Ecology Letters 22: 2130–2140. 31625279 10.1111/ele.13400

[CIT0023] Liang X , Hyyppä J. 2013. Automatic stem mapping by merging several terrestrial laser scans at the feature and decision levels. Sensors 13: 1614–1634. 23353143 10.3390/s130201614 PMC3649416

[CIT0024] Liang X , Kukko A, Kaartinen H, et al. 2014. Possibilities of a personal laser scanning system for forest mapping and ecosystem services. Sensors 14: 1228–1248. 24434879 10.3390/s140101228 PMC3926612

[CIT0025] Liang X , Kankare V, Hyyppä J, et al. 2016. Terrestrial laser scanning in forest inventories. ISPRS Journal of Photogrammetry and Remote Sensing 115: 63–77.

[CIT0026] Liang X , Hyyppä J, Kaartinen H, et al. 2018. International benchmarking of terrestrial laser scanning approaches for forest inventories. ISPRS Journal of Photogrammetry and Remote Sensing 144: 137–179.

[CIT0027] Lin Y , Jaakkola A, Hyyppä J, Kaartinen H. 2010. From TLS to VLS: biomass estimation at individual tree level. Remote Sensing 2: 1864–1879.

[CIT0028] Liu G , Wang J, Dong P, Chen Y, Liu Z. 2018. Estimating individual tree height and diameter at breast height (DBH) from terrestrial laser scanning (TLS) data at plot level. Forests 9: 398.

[CIT0029] Maas H-G , Bienert A, Scheller S, Keane E. 2008. Automatic forest inventory parameter determination from terrestrial laser scanner data. International Journal of Remote Sensing 29: 1579–1593.

[CIT0030] Metz JÔ , Seidel D, Schall P, Scheffer D, Schulze ED, Ammer C. 2013. Crown modeling by terrestrial laser scanning as an approach to assess the effect of aboveground intra- and interspecific competition on tree growth. Forest Ecology and Management 310: 275–288.

[CIT0031] Murphy GE , Acuna MA, Dumbrell I. 2010. Tree value and log product yield determination in radiata pine (*Pinus radiata*) plantations in Australia: comparisons of terrestrial laser scanning with a forest inventory system and manual measurements. Canadian Journal of Forest Research 40: 2223–2233.

[CIT0032] Olofsson K , Holmgren J, Olsson H. 2014. Tree stem and height measurements using terrestrial laser scanning and the RANSAC algorithm. Remote Sensing 6: 4323–4344.

[CIT0033] Raumonen P , Kaasalainen M, Åkerblom M, et al. 2013. Fast automatic precision tree models from terrestrial laser scanner data. Remote Sensing 5: 491–520.

[CIT0034a] RIEGL . 2012. Compact mobile laser scanning system: datasheet RIEGL VMX-250. https://www.riegl.com (Accessed 20 September 2020).

[CIT0034] R Core Team . 2017. R: A language and environment for statistical computing. Vienna, Austria: R Foundation for Statistical Computing. https://www.R-project.org/.

[CIT0035] Rusu RB , Cousins S. 2011. 3D is here: point cloud library (PCL). Proceedings - IEEE International Conference on Robotics and Automation. doi: 10.1109/ICRA.2011.5980567.

[CIT0036] Schilling A , Schmidt A, Maas H-G. 2011. Automatic tree detection and diameter estimation in terrestrial laser scanner point clouds. Proceedings of the 16th Computer Vision Winter Workshop, Mitterberg, Austria, 75–83.

[CIT0036a] Seidel D , Leuschner C, Müller A, Krause B. 2011. Crown plasticity in mixed forests – quantifying asymmetry as a measure of competition using terrestrial laser scanning. Forest Ecology and Management 261: 2123–2132.

[CIT0037] Simonse M , Aschoff T, Spiecker H, Thies M. 2003. Automatic determination of forest inventory parameters using terrestrial laserscanning. In: Proc. of the ScandLaser Scientific Workshop on Airborne Laser Scanning of Forests, Umea, Sweden, 251–257. http://www.natscan.uni-freiburg.de/suite/pdf/030916_1642_1.pdf

[CIT0039] Tansey K , Selmes N, Anstee A, Tate NJ, Denniss A. 2009. Estimating tree and stand variables in a Corsican Pine woodland from terrestrial laser scanner data. International Journal of Remote Sensing 30: 5195–5209.

[CIT0040] Trochta J , Kruček M, Vrška T, Kraâl K. 2017. 3D Forest: an application for descriptions of three-dimensional forest structures using terrestrial LiDAR. PLoS One 12: e0176871. 28472167 10.1371/journal.pone.0176871 PMC5417521

[CIT0041] Wang J , Lindenbergh R, Menenti M. 2018. Scalable individual tree delineation in 3D point clouds. Photogrammetric Record 33: 315–340.

[CIT0042] Wang P , Li R, Bu G, Zhao R. 2019. Automated low-cost terrestrial laser scanner for measuring diameters at breast height and heights of plantation trees. PLoS One 14: e0209888. 30653532 10.1371/journal.pone.0209888 PMC6336300

[CIT0043] Wang Y , Chen Q, Zhu Q, Liu L, Li C, Zheng D. 2019 *a*. Laser scanning applications and key techniques over urban areas. Remote Sensing 11: 1540.

[CIT0044] Wang Y , Lehtomäki M, Liang X, et al. 2019 *b*. Is field-measured tree height as reliable as believed – a comparison study of tree height estimates from field measurement, airborne laser scanning and terrestrial laser scanning in a boreal forest. ISPRS Journal of Photogrammetry and Remote Sensing 147: 132–145.

[CIT0045] Wang Y , Kukko A, Hyyppä E, et al. 2021. Seamless integration of above- and under-canopy unmanned aerial vehicle laser scanning for forest investigation. Forest Ecosystems 8: doi: 10.1186/s40663-021-00290-3.

[CIT0046] Wu B , Yu B, Yue W, et al. 2013. A voxel-based method for automated identification and morphological parameters estimation of individual street trees from mobile laser scanning data. Remote Sensing 5: 584–611.

