# Supplementary material for: Automatic extraction and measurement of individual trees from mobile laser scanning point clouds of forests
Source: Ann Bot. 2021 Jul 7;128(6):787–804. doi: 10.1093/aob/mcab087 (PMC8557376; doi:10.1093/aob/mcab087)
Supplement: mcab087_suppl_Supplementary_Tables [file mcab087_suppl_supplementary_tables.docx]

Appendix

**Appendix Tables**

Table. A1. Characteristics of ten sample plots sorted by distance from the MLS trajectory (forest track). Data from forest inventories of the Forestry Office of the city of Lübeck. For clarity, the tree species have been abbreviated as follows*: F. sylvatica (Fasy), C. betulus (Cabe), Qu. robur (Quro), A. pseudoplatanus (Acps), F. excelsior (Frex), L. decidua (Lade), P. avium (Prav), A. platanoides (Acpl)* and *B. pendula (Bepe).*

| **Group** | **Plot name** | **Species composition** | **Age [years]** | **Tree density (DBH**≥**7 cm) [trees/ha]** | **Mean**  **DBH**  **[cm]** | **Mean**  **tree height**  **[m]** |
| --- | --- | --- | --- | --- | --- | --- |
| **A [0 m-20 m]** | **1** | 76.5 % *Fasy*  17.6 % *Quro*  2.9 % *Cabe*  2.9 % *Bepe* | 197 | 212 | 39.88 | 22.37 |
|  | **2** | 82.0 % *Fasy*  11.0 % *Quro*  7.0 % *Cabe* | 87 | 623 | 22.65 | 19.95 |
|  | **3** | 50.0 % *Fasy*  20.0 % *Quro*  26.0 % *Cabe*  2.0 % *Frex*  2.0 % *Acps* | 157 | 312 | 27.79 | 19.13 |
| **B [20 m-40 m]** | **1** | 80.4 % *Fasy*  4.3 % *Quro*  2.2 % *Cabe*  4.3 % *Frex*  8.7 % *Acps* | 168 | 287 | 33.33 | 23.00 |
|  | **2** | 2.4 % *Fasy*  18.3 % *Quro*  68.3 % *Cabe*  9.8 % *Frex*  1.2 % *Acpl* | 125 | 511 | 25.65 | 21.62 |
|  | **3** | 51.1 % *Fasy*  8.5 % *Quro*  33.3 % *Cabe*  1.4 % *Prav*  5.7 % *Frex* | 124 | 879 | 18.93 | 17.95 |
|  | **4** | 11.9 % *Fasy*  33.3 % *Quro*  26.2 % *Cabe*  28.6 % *Acps* | 144 | 262 | 38.33 | 24.46 |
| **C [40 m-60 m]** | **1** | 91.6 % *Fasy*  1.9 % *Quro*  2.8 % *Lade*  3.7 % *Acps* | 58 | 667 | 20.54 | 20.64 |
|  | **2** | 77.8 % *Fasy*  22.2 % *Quro* | 102 | 393 | 35.33 | 27.50 |
|  | **3** | 93.8 % *Fasy*  5.6 % *Lade*  0.6 % *Acps* | 53 | 997 | 18.54 | 19.46 |

Table. A2. Percentage rates of the over- and undersegmentation of all plots.

| **Group** | **Plot name** | **Undersegmentation [%]** | **Oversegmentation [%]** |
| --- | --- | --- | --- |
| **A** | **1** | 43.3 | 18.0 |
|  | **2** | 50.0 | 26.5 |
|  | **3** | 43.5 | 23.0 |
| **B** | **1** | 60.5 | 11.1 |
|  | **2** | 42.6 | 20.3 |
|  | **3** | 57.6 | 13.8 |
|  | **4** | 63.1 | 25.3 |
| **C** | **1** | 83.9 | 5.2 |
|  | **2** | 79.7 | 4.9 |
|  | **3** | 88.1 | 13.4 |
|  | **Mean** | **61.2** | **16.1** |

**Appendix Figures**

Fig. A1. Number of automatically detected MLS trees and undetected manually added trees based on MLS data divided according to distance from trajectory. The percentages given are the detection rates for the automatic tree detection and the manual added trees. In addition, the incorrectly classified (light blue) and falsly added (light orange) trees were also plotted. Please note that the true number of trees is larger than the MLS detected trees.

Fig. A2. Automatically segmented MLS plots compared to the semi-automatically segmented TLS plots of group B. Individual trees are indicated by different colours and only the corresponding trees are displayed. For printing purposes the same visualisation parameters were used for both data sets.

Fig. A3. Automatically segmented MLS plots compared to the semi-automatically segmented TLS plots of group C. Individual trees are indicated by different colours and only the corresponding trees are displayed. For printing purposes the same visualisation parameters were used for both data sets.
