# Supplementary figures and images for: Automatic extraction and measurement of individual trees from mobile laser scanning point clouds of forests
Source: Ann Bot. 2021 Jul 7;128(6):787–804. doi: 10.1093/aob/mcab087 (PMC8557376; doi:10.1093/aob/mcab087)

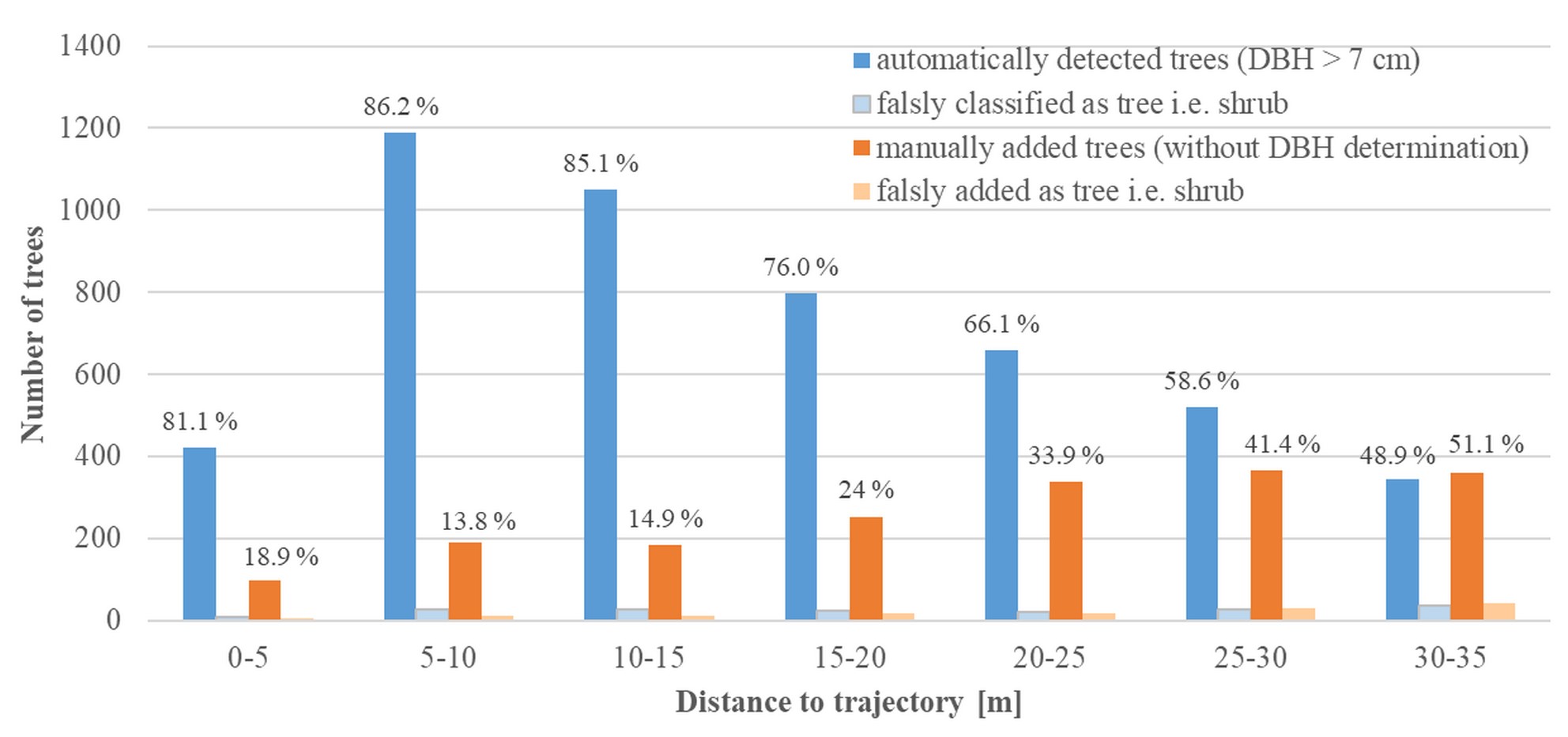

Supplement: mcab087_suppl_Supplementary_Figure_S1 [file mcab087_suppl_supplementary_figure_s1.jpeg]

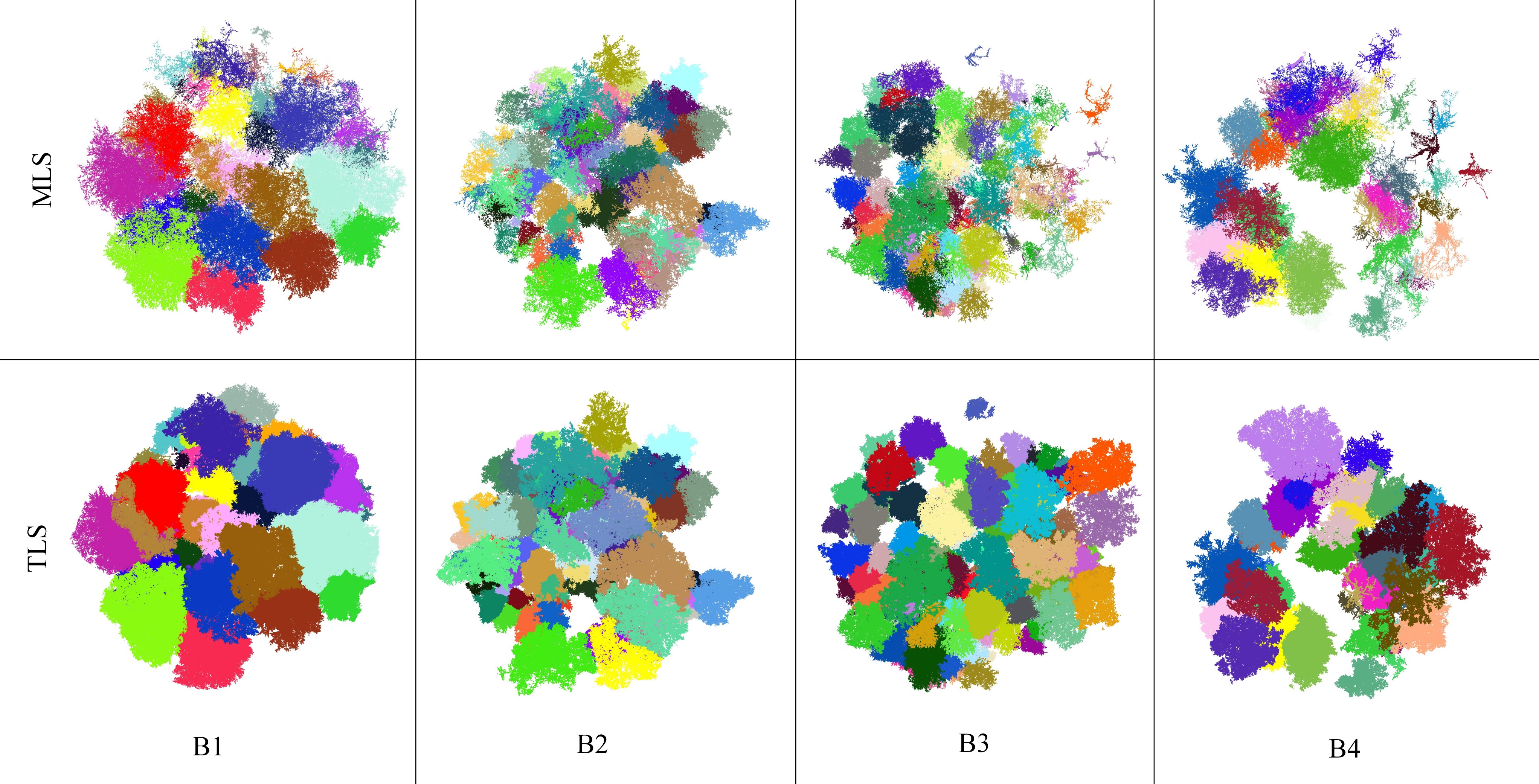

Supplement: mcab087_suppl_Supplementary_Figure_S2 [file mcab087_suppl_supplementary_figure_s2.jpeg]

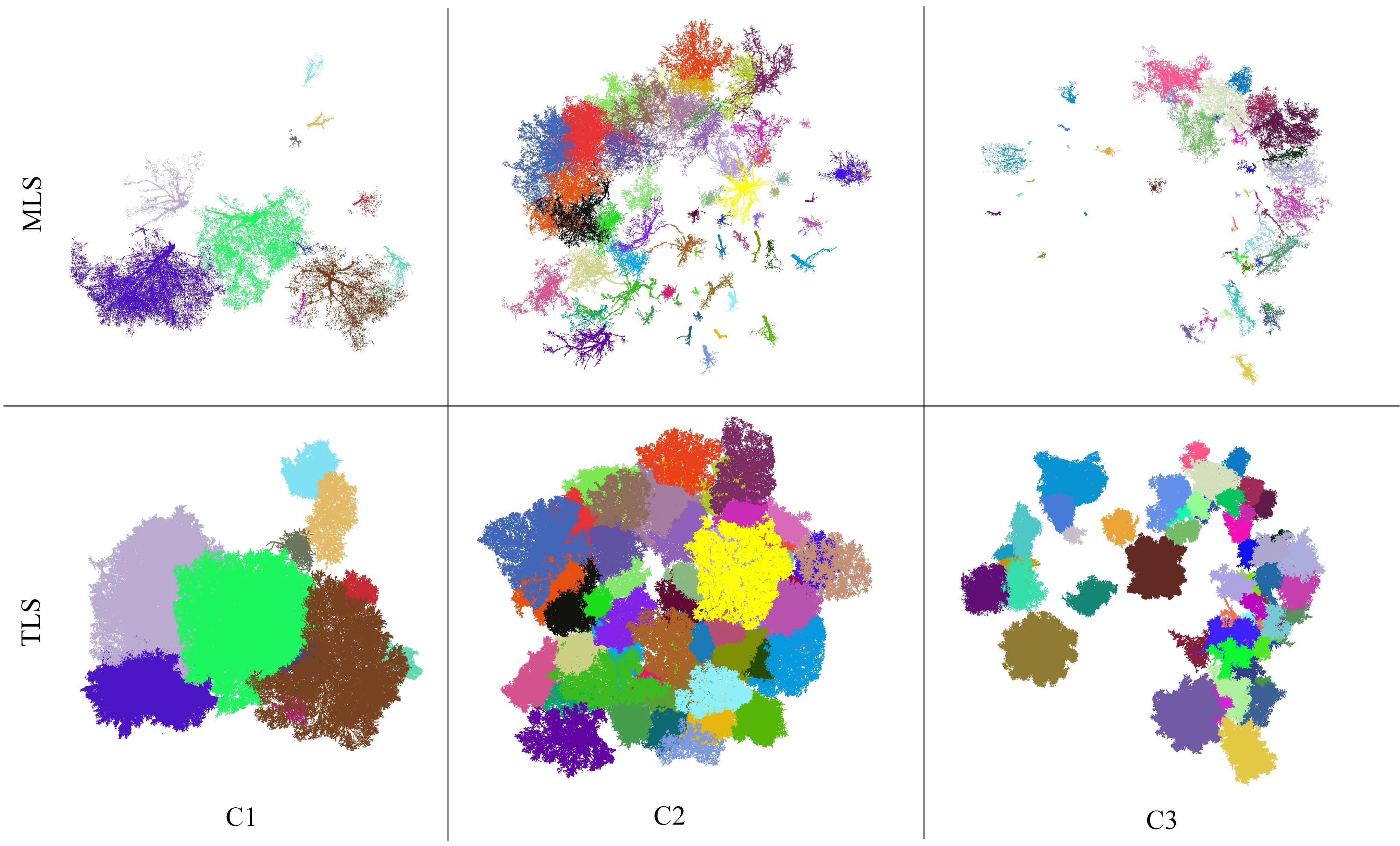

Supplement: mcab087_suppl_Supplementary_Figure_S3 [file mcab087_suppl_supplementary_figure_s3.jpeg]
